# Multicenter retrospective study of the first robotic surgeries in selected Brazilian centers

**DOI:** 10.1590/1806-9282.20260174

**Published:** 2026-08-10

**Authors:** Aline Akel Ferruccio, Murilo de Almeida Luz, Wagner Eduardo Matheus, Ubirajara Ferreira

**Affiliations:** 1 Universidade Estadual de Campinas – Campinas (SP), Brazil.

**Keywords:** Robotic-assisted surgery, Medical education, Technology, Minimally invasive surgical procedures

## Abstract

**OBJECTIVE:**

Advances in robotics have established the surgical modality approach as gold standard for several procedures. The aim of this study was to present a demographic analysis of the first robotic surgeries performed in selected Brazilian centers over a 5-year period, with insights on the evolution of this technology.

**METHODS:**

This retrospective cohort study analyzed data from 16 Brazilian hospitals equipped with robotic platforms between 2016 and 2020. Surgical procedures were evaluated according to specialty, primary surgeon, presence of a proctor, operating room time, console time, hospital, and year of performance. Surgeon experience was estimated based on time since medical graduation and categorized. Statistical analysis was performed using Generalized Equations.

**RESULTS:**

A total of 4,363 surgeries were analyzed, with 80% performed in São Paulo state. General surgery, urology, and gynecology accounted for most procedures. A progressive reduction in console time was observed over the years, considering procedures in general, specific procedure time-trend analysis and within high-volume surgeons, but no statistical evidence was drawn and findings are exploratory. This study is limited by its retrospective design, lack of standardized data regarding individual training pathways, and absence of clinical outcome measures. Therefore, it is a descriptive audit of case volumes and operative times, without interpretation of surgical quality or patient safety.

**CONCLUSION:**

Robotic surgery has expanded rapidly in Brazil, with concentration in the Southeast region. Procedural volume and structured training are primary factors associated with improved performance. Early integration of robotic surgery into surgical education may optimize learning curves and surgeon proficiency.

## INTRODUCTION

Robotic surgery has evolved into a widely adopted surgical approach, offering enhanced dexterity, three-dimensional visualization, and improved ergonomics. These advantages have facilitated the execution of complex procedures and expanded robotic applications across multiple surgical specialties^1,2^.

Globally, more than 17 million robotic procedures have been performed using da Vinci systems across over 9,900 platforms^3^. In Brazil, robotic surgery was introduced in 2008 and has expanded rapidly, prompting national regulations addressing surgeon qualification and structured training^4,5^.

Despite technological advances, variability persists in surgeon experience, procedural volume, and learning curves. Understanding the relationship between surgeon profile and operative performance is essential to optimize training strategies. This study aimed to analyze the demographic profile of early robotic surgery in Brazil, with insights on the evolution of this technology.

## METHODS

**Figure 1:**
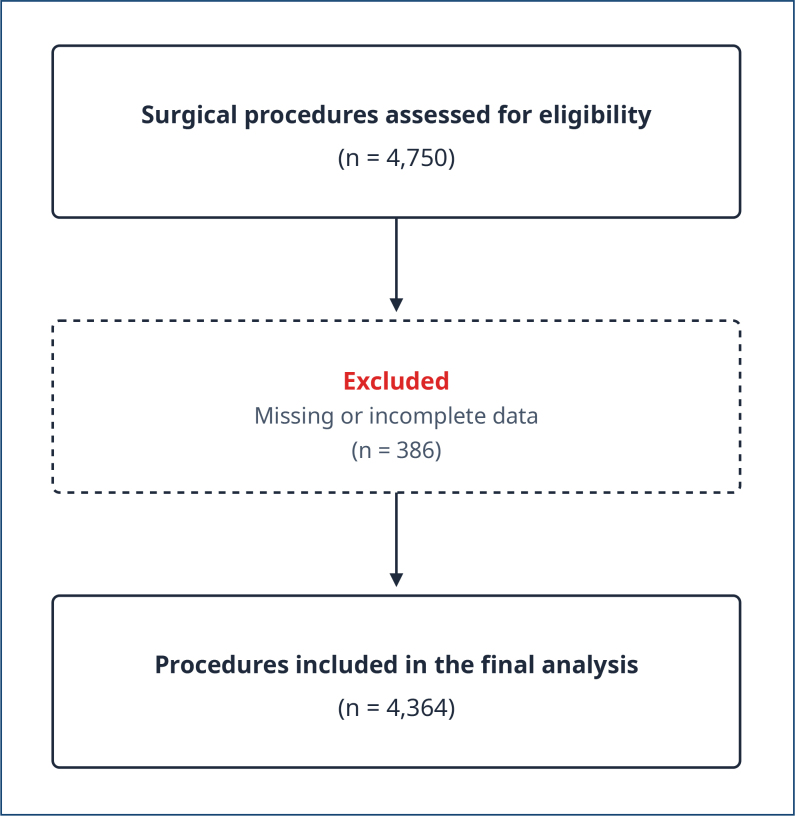
Flow diagram of patient selection.

This retrospective cohort study included 4,363 robotic procedures performed between 2016 and 2020 in 16 Brazilian hospitals equipped with Intuitive Surgical da Vinci platforms, with concentration in the Southeast region. Adult patients undergoing robot-assisted surgery were included. Procedures aborted before robotic docking and cases with incomplete data were excluded (Figure 1).

Collected variables included procedure type, specialty, primary surgeon, proctor presence, operating room time, console time, hospital, and year. Operating room time included everything that occurred between the patient's admission and leaving for recovery, regarding anesthesia, positioning, docking and draping. Console time was defined as operative time after robotic docking. Surgeon experience was categorized based on years since medical graduation, considering it was not possible to level the robotic training pathway or the volume of surgeries per surgeon.

Data was analyzed with descriptive statistics. For the comparison of parameters in relation to the variables of interest, the Generalized Estimating Equations method was used. Estimates were calculated using maximum likelihood estimation in order to account for differences in the number of procedures performed by each professional. The models were adjusted assuming a normal distribution and an identity link function. An exchangeable working correlation structure was adopted to account for dependency and correlation between observations. A significance level of 5% was considered.

## RESULTS

A total of 4,363 robotic surgeries performed using the da Vinci system between 2016 and 2020 were retrospectively analyzed across 16 accredited hospitals in Brazil. Of these centers, 10 were in the Southeast region, 3 in the Central-West, and 3 in the Northeast. State-level distribution showed that São Paulo accounted for 80% of all procedures, followed by Rio de Janeiro (7%), Pernambuco (5.5%), the Federal District (3.8%), Bahia (2.2%), and Sergipe (1.5%).

**Figure 2:**
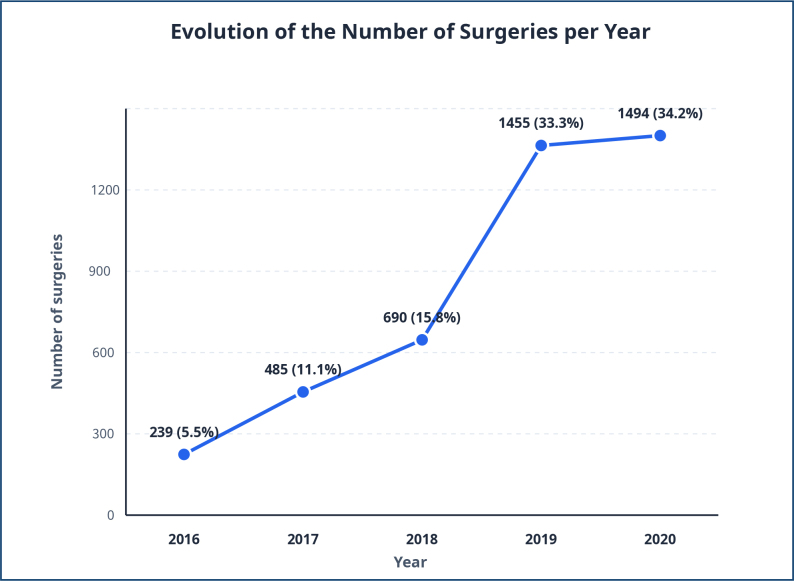
Number of robotic surgeries per year.

Annual distribution demonstrated progressive growth: 239 surgeries (5.5%) in 2016, 485 (11.1%) in 2017, 690 (15.8%) in 2018, 1,455 (33.3%) in 2019, and 1,494 (34.2%) in 2020 (Figure 2).

Seven surgical specialties utilized robotic platforms, with general surgery accounting for 1,719 procedures (39.4%), urology for 1,371 (31.4%), gynecology for 1,127 (25.8%), thoracic surgery for 129 (3%), and head and neck surgery, otorhinolaryngology, and plastic surgery comprising the remaining 3%. The most frequently performed procedures were radical prostatectomy, endometriosis repair, hernia repair, bariatric surgery, and hysterectomy.

Mean operating room time was 286.8 min [standard deviation (SD) 113.4], with a median of 270 min. Mean console time was 154.2 min (SD 97.2), with a median of 135 min. When console time was analyzed across the study period without stratification by specialty or procedure, mean times were 184.2 min (SD 97.2) in 2016; 184.2 min (SD 91.8) in 2017; 182.4 min (SD 129.6) in 2018; 138.6 min (SD 87.6) in 2019; and 141.6 min (SD 83.4) in 2020. A significant reduction in operative time was observed between 2017 and 2019, 2017 and 2020, 2018 and 2019, and 2018 and 2020 (p=0.0262).

**Figure 3:**
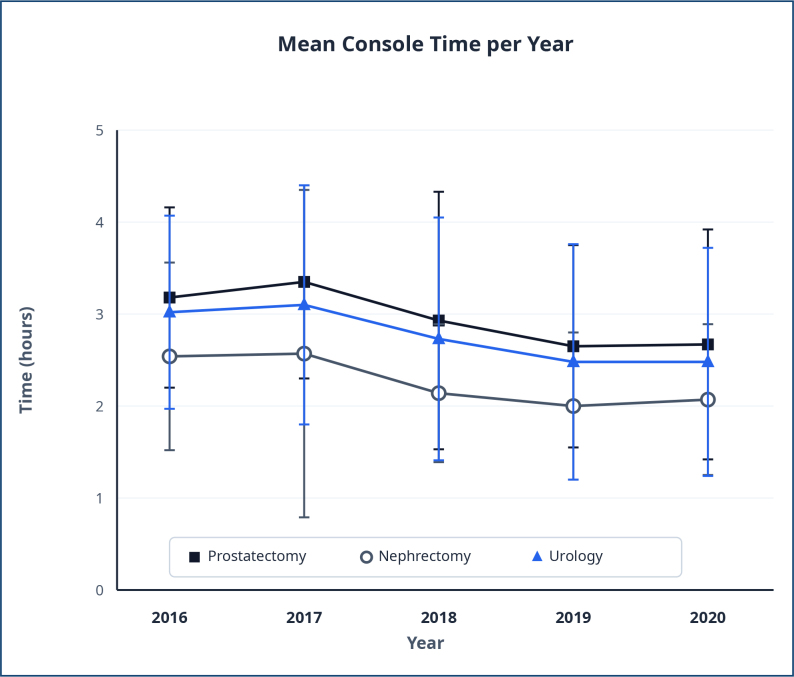
Mean console time per year.

Console time trends were further evaluated by specialty and procedure. Mean console time was similar among the most active specialties, while variability among procedures reflected differences in procedural complexity. Urology demonstrated a progressive reduction in console time and specific procedure time trend analysis were conducted (Figure 3).

Radical prostatectomies mean console times were 190.2 min (SD 59.4) in 2016; 237.0 min (SD 61.8) in 2017; 175.8 min (SD 84.0) in 2018; 158.4 min (SD 66.6) in 2019; and 159.6 min (SD 75.6) in 2020. Nephrectomy mean console times were 151.8 min (SD 61.8) in 2016; 153.6 min (SD 113.4) in 2017; 128.4 min (SD 43.2) in 2018; 119.4 min (SD 96.6) in 2019; and 124.2 min (SD 66.0) in 2020. These comparisons did not reach statistical significance (p=0.1241 for urology; 0.1550 for prostatectomy; 0.5466 for nephrectomy). They are exploratory and hypothesis-generating.

General surgery demonstrated a reduction in mean console time from 210.0 min (SD 108.0) in 2016 to 132.6 min (SD 89.4) in 2020, while gynecology showed a reduction from 173.4 min (SD 103.8) to 150.0 min (SD 81.6) over the same period. However, in these surgical specialties, differences between console time in specific procedures were not addressed, limiting the interpretations of the time-trend analysis. These trends did not reach statistical significance (p=0.1531 and p=0.1841, respectively).

Of the total procedures, 3,695 (84.7%) were performed without proctor supervision. Console time was significantly longer in proctored surgeries (162.6 min; SD 83.4) compared with non-proctored procedures (152.4 min; SD 99.6) (p=0.0134).

**Figure 4:**
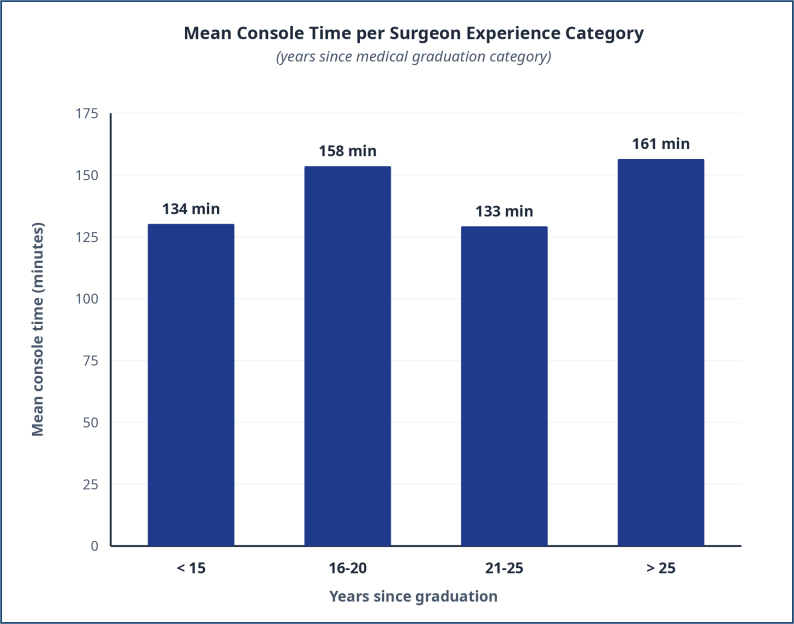
Mean console time (min) per surgeon experience category (years/graduation).

A total of 384 surgeons performed the procedures analyzed. Mean time since graduation was 29.5 years (SD 9.69), with a median of 28 years (range 8–53). Proctors had a shorter mean time since graduation (25.1 years; SD 8.82) compared to the full group of surgeons in the study (29.5 years; SD 9.69). Professionals with more than 25 years since graduation accounted for 41.4% of the cohort and performed 61.8% of all procedures. No statistically significant association was observed between surgeon experience category and console time (p=0.1658), although shorter mean console times were observed among surgeons with fewer years since graduation (Figure 4).

The five most active surgeons within urology, general surgery, and gynecology also demonstrated a progressive reduction in console time over the years observed in this demographical study.

## DISCUSSION

The present study provides a comprehensive overview of the early expansion of robotic surgery in Brazil, highlighting its rapid growth, concentration in large centers and the Southeast region. The specialties with greater surgical volume were general surgery, urology, and gynecology^6^. Urology accounted for more than 30% of procedures, consistent with its established role in robotic surgery and the robust evidence supporting its oncologic and functional outcomes^7^.

A progressive reduction in console time was observed over the years, considering procedures in general, specific procedure time-trend analysis and within high-volume surgeons, but no statistical evidence was drawn in this demographical study and the results are exploratory Procedural volume is reasonable as a key determinant of performance improvement, reinforcing previous studies regarding surgical training. In contrast, longer professional experience, measured as time since medical graduation, did not consistently correlate with shorter operative times. Considering that the robotic-specific experience was not an available data, we acknowledge that as a major limitation to this study and its findings. However, literature suggests that younger surgeons who are more familiar to digital interfaces and have early exposure to robotic platforms could have a more facilitate skill acquisition^8^.

The presence of a proctor was associated with longer console times, likely reflecting the supervisory role during early phases of training or more complex cases. This finding is descriptive, once the study was unable to analyze the complexity of each supervised surgery or surgeon experience when proctors were present, and might contain selection bias within the reachable information.

Advances in structured training pathways, simulation, and credentialing have contributed to improved learning curves and broader dissemination of robotic surgery in Brazil^9–11^. Nevertheless, disparities in access to platforms and formal training remain challenges, particularly during residency training. Simulation-based education and supervised clinical exposure continue to play complementary roles in achieving technical proficiency^12–16^.

This study is limited by its retrospective design, lack of standardized data regarding individual training pathways, and absence of clinical outcome measures. Therefore, it is a descriptive audit of case volumes and operative times, without interpretation of surgical quality or patient safety. Nonetheless, the findings provide relevant insights into training and development in robotic surgery, as well as the evolution of this technology within the Brazilian healthcare system.

## CONCLUSION

Robotic surgery has grown exponentially in Brazil, with concentration in the Southeast region. Procedural volume and structured training are primary factors associated with improved performance. Early integration of robotic surgery into surgical education may optimize learning curves and surgeon proficiency.

## Data Availability

The datasets generated and/or analyzed during the current study are available from the corresponding author upon reasonable request.
